# Determining the factors affecting the distribution of *Muscari latifolium*, an endemic plant of Turkey, and a mapping species distribution model

**DOI:** 10.1002/ece3.2766

**Published:** 2017-01-23

**Authors:** Hatice Yilmaz, Osman Yalçın Yilmaz, Yaşar Feyza Akyüz

**Affiliations:** ^1^ Ornamental Plants Cultivation ProgramVocational School of ForestryFaculty of ForestryIstanbul UniversityIstanbulTurkey; ^2^ Department of Forest EngineeringFaculty of ForestryIstanbul UniversityIstanbulTurkey

**Keywords:** abiotic factors, biotic factors, boosted regression modeling, bulbous plant, species distribution modeling

## Abstract

Species distribution modeling was used to determine factors among the large predictor candidate data set that affect the distribution of *Muscari latifolium*, an endemic bulbous plant species of Turkey, to quantify the relative importance of each factor and make a potential spatial distribution map of *M. latifolium*. Models were built using the Boosted Regression Trees method based on 35 presence and 70 absence records obtained through field sampling in the Gönen Dam watershed area of the Kazdağı Mountains in West Anatolia. Large candidate variables of monthly and seasonal climate, fine‐scale land surface, and geologic and biotic variables were simplified using a BRT simplifying procedure. Analyses performed on these resources, direct and indirect variables showed that there were 14 main factors that influence the species’ distribution. Five of the 14 most important variables influencing the distribution of the species are bedrock type, *Quercus cerris* density, precipitation during the wettest month, *Pinus nigra* density, and northness. These variables account for approximately 60% of the relative importance for determining the distribution of the species. Prediction performance was assessed by 10 random subsample data sets and gave a maximum the area under a receiver operating characteristic curve (AUC) value of 0.93 and an average AUC value of 0.8. This study provides a significant contribution to the knowledge of the habitat requirements and ecological characteristics of this species. The distribution of this species is explained by a combination of biotic and abiotic factors. Hence, using biotic interaction and fine‐scale land surface variables in species distribution models improved the accuracy and precision of the model. The knowledge of the relationships between distribution patterns and environmental factors and biotic interaction of *M. latifolium* can help develop a management and conservation strategy for this species.

## 1 Introduction

Endemic species grow naturally in restricted geographic ranges, and specific habitats and are prone to become endangered under changing environmental conditions and other threats. They also have a great tendency to become extinct if they are both rare and endemic (Işık, 2011; Lomba et al., 2010; Marcer, Sáez, Molowny‐Horas, Pons, & Pino, 2013). Sustainable management practices and the preservation of endemic and rare plants are essential for the conservation of global biodiversity because these plants are important not only for local regions but also for global biodiversity. Therefore, endemic species are important targets for global conservation efforts (Myers, Mittermeier, Mittermeier, da Fonseca, & Kent, 2000).

**Figure 1:**
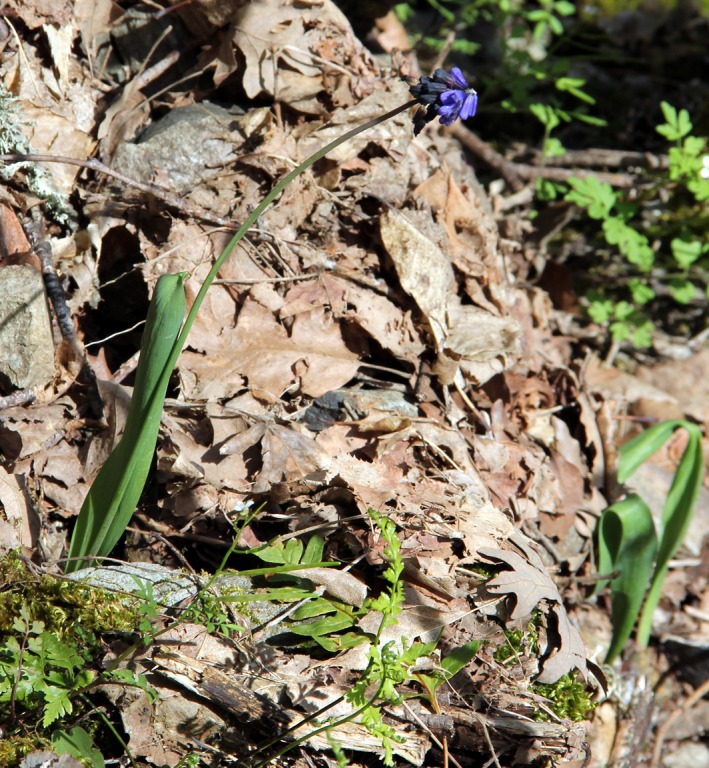
*Muscari latifolium*. Photographed in Gönen Dam watershed, Turkey, April 2013

*Muscari* is a genus of 46 species, distributed across Europe, Asia, and North Africa (Govaerts, Zonneveld, & Zona, 2015). Thirty‐three of these species occur naturally in Turkey (Davis & Stuart, 1984; Demirci, Özhatay, & Koçyiğit, 2013; Güner, 2012; Pirhan, Yıldırım, & Altıoğlu, 2014; Yıldırım, 2015). *Muscari latifolium* J. Kirk. (Asparagaceae) (Figure 1) is an endemic bulbous plant species of Turkey with a highly local distribution in western, inner western, and southwestern Turkey, Balıkesir–Çanakkale Kazdağı Mountains, Kütahya Murat Mountain, and Antalya Akseki at altitudes between 1,100 and 1,800 m in *Pinus nigra* J.F. Arnold and *Pinus sylvestris* L. forests (Davis & Stuart, 1984). Bulbs are solitary, ovoid, and 1.5–3 cm in diameter. Leaves are usually solitary, and two are found in rare cases, erect, broadly linear, lanceolate, 7–30 cm long, and 10–30 mm wide. Flowers are carried on a scape longer than the leaves. Inflorescences are racemes 1.5–6 cm long and consist of both fertile and sterile flowers. Sterile flowers are pale violet to light blue, 4–8 mm long, and located at the top of the raceme, whereas fertile flowers are dark violet to black, 5–6 mm long, and located at the bottom of the raceme. Fruit is a capsule 7–8 mm in size (Davis & Stuart, 1984). The species, which its flowering period is in April and May, are being used as ornamental plants (Bryan & Hort, 2002) and are usually propagated from seeds (Wraga & Placek, 2009). *Muscari latifolium* is easy to detect even outside the flowering season because of its broad leaves. It prefers lime and slightly acidic loamy soil with potassium, high phosphorus content, and rich organic matter (Hopa, Tümen, Sevindik, & Selvi, 2013).

It is important to know the distribution, ecological traits, and population structure of endemic plant species to manage them in a sustainable manner and to develop effective conservation strategies for them. Determining the entire distribution area of a plant species is neither feasible nor realistic merely by navigating through the area without sampling. Furthermore, despite the fact that the area is well sampled, the organism will be present outside the sampling plots. However, species distribution models (SDMs) give us the ability to predict the distribution of the species across a landscape or within a certain time frame (Elith & Leathwick, 2009; Guisan & Thuiller, 2005; Peterson, 2006). SDMs are suitable tools for understanding the realized species distribution and for estimating the species’ potential distribution for endemic and rare species in well‐surveyed areas (Marcer et al., 2013; Williams et al., 2009).

In SDMs, presence–absence, presence‐only, or abundance data are used to predict species distribution. Presence–absence data provide valuable information about the availability and prevalence of species in the research area and allow for more ecologically realistic predictions to be made (Elith & Leathwick, 2009; Phillips, Dudik, Elith, Graham, & Lehmann, 2009). Either the presence–absence or the abundance of vascular plants is affected by three main groups of factors: direct, indirect, and resource gradients (Austin, 2002; Franklin, 2009; Guisan & Zimmermann, 2000). Additionally, the occurrence of *a* herbaceous plant species in a forest can be affected by overstory and understory species, canopy closure, and disturbances such as human or animal activities. These biotic factors are difficult to measure and analyze, and they are often ignored in SDMs, even when they are necessary to make realistic predictions (Wisz et al., 2013). *M. latifolium* usually grows in forest understories. The occurrence of this species might be affected by overstory tree species, development stage of trees, canopy, and characteristics of shrub layer. Moreover, the distances of sample plots to the nearest settlement area may cause indirect human and livestock disturbance effects.

In many species distribution modeling studies, the model is established using selected variables based on the accumulated ecological literature (Porfirio et al., 2014). However, the terrain effects on plant distribution can be explained better by making use of variables derived from digital elevation models (DEMs). These are variables that may have indirect effects on the distribution and abundance of plants. Additionally, annual climate variables are usually used in plant modeling studies. However, climate data should be evaluated on a monthly and seasonal basis. Because herbaceous plants have different life cycles and many different characteristics such as root depth and stem structure, they are more affected than trees by extreme climate values, short term, and seasonal fluctuations (Brovkin, 2002). There have been limited attempts to use fine‐scale DEM‐derived variables and monthly climatic data in species distribution studies.

A variety of methods, such as BIOCLIM (Nix, 1986), MaxEnt (Phillips, Anderson, & Schapire, 2006), DOMAIN (Carpenter, Gillison, & Winter, 1993), GAM (Hastie & Tibshirani, 1990), GLM (McCullough & Nelder, 1989), and random forest (Breiman, 2001), can be used in SDMs. However, this study focuses on identifying species–environment relationships and on estimating the realistic potential distribution area of the species, not on comparing the results of different modeling methods. The aim of this study is to determine the influence of climatic, land surface, geologic, and biotic variables on the distribution of *M. latifolium*. The study also aims to evaluate the prediction power of models fitted with the “Boosted Regression Trees” (BRT) method based on presence/absence data and a large environmental variable data set. We also summarize the relative importance of predictor variables. The BRT method was preferred in this study because it provides highly accurate predictions of species distribution models and variable shrinkage (Elith, Leathwick, & Hastie, 2008), and it is more sensitive to local site conditions (Falk & Mellert, 2011).

## 2 Materials and Methods

### 2.1 Study area

**Figure 2:**
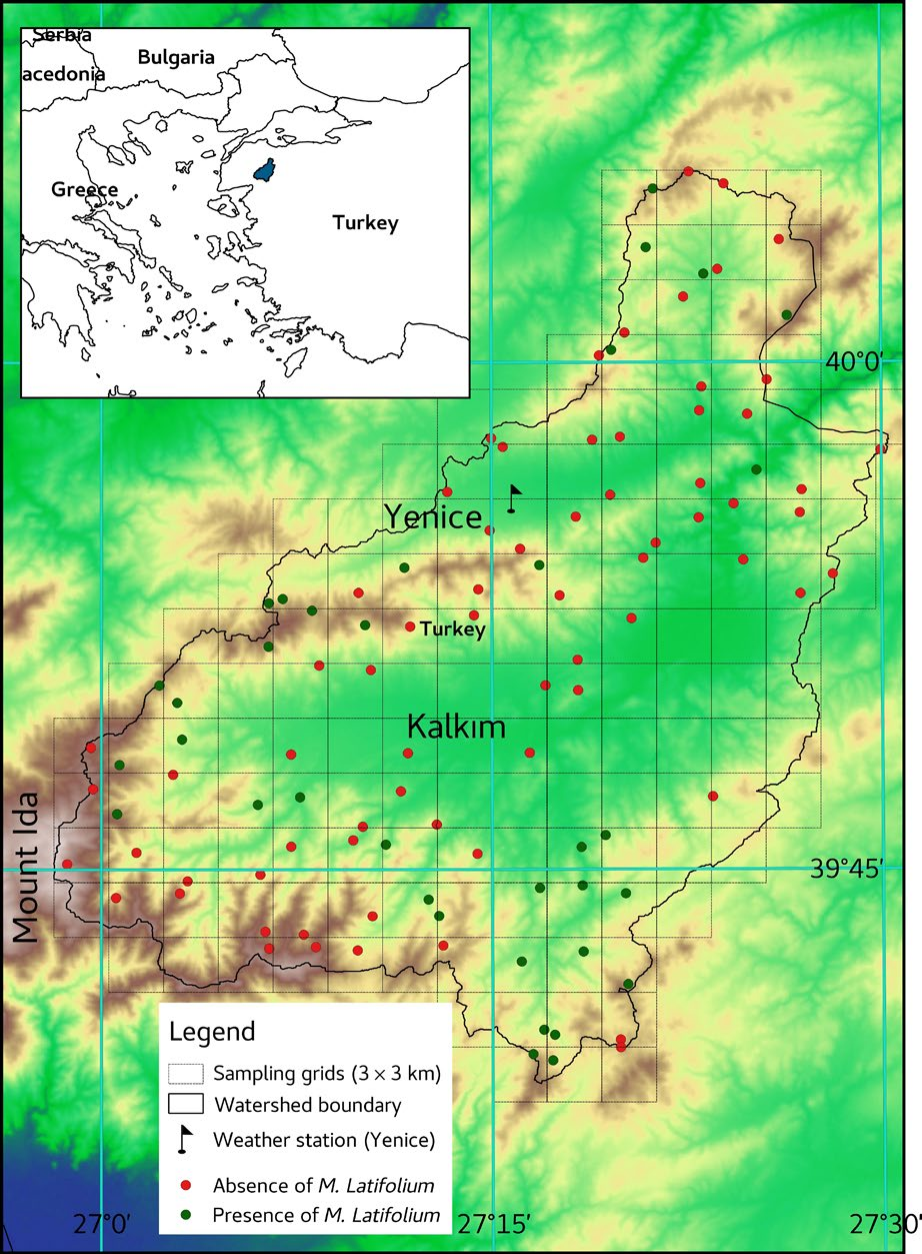
Location of the studied area (filled blue) and distribution of *Muscari latifolium* incidence on a 3 × 3 km grid in Gönen Dam watershed (Turkey)

The study was conducted in the Gönen Dam watershed area, which covers 113,700 ha and ranges from 90 to 1,400 m a.s.l. (Figure 2). According to long‐term data from the nearest meteorological station located in the Yenice Province, long‐term average of annual total precipitation is 847.3 mm, and the mean annual temperature is 12.8°C. The Gönen Dam watershed area is located in the northeast Kazdağı Mountains (formerly known as Ida Mountain) in West Anatolia (26.960‐27.540°E, 39.640‐40.100°N). The Kazdağı Mountains consist of several mountain peaks and plateaus and were classified as an IPA (important plant area) not only for Turkey but also for Europe because they contain a high numbers of endemic and rare plant species (Özhatay & Özhatay, 2005). Forests in the Kazdağı Mountains are composed of both pure and mixed conifer and broadleaf trees, such as *Pinus nigra* J.F. Arnold subsp. *pallasiana* (Lamb.) Holmboe, *Pinus brutia* Ten., *Abies nordmanniana* (Steven) Spach subsp. *equi‐trojani* (Asch. & Sint. ex Boiss.) Coode & Cullen, *Quercus* sp., *Fagus orientalis* Lipsky, maquies, and thickets.

### 2.2 Species data

To obtain a representative sample (Araujo & Guisan, 2006) of *M. latifolium* occurrence in the study area, it was systematically divided into 3 km × 3 km grids. Then, a 20 m × 20 m quadrat was randomly assigned in each grid, excluding agricultural and residential areas. To avoid edge effects, the quadrats were assigned at least 50 m away from roads. A total of 105 plots in the study area were in managed forests (Figure 2). Therefore, the species incidence consists of 35 presence and 70 absence records. *M. latifolium* was detected at altitudes ranging from 189 to 885 m in the study area, although the reported range was between 1,100 and 1,800 m (Davis & Stuart, 1984).

This study uses *M. latifolium* presence–absence data as the response variable. As suggested by Lobo, Jiménez‐Valverde, and Hortal (2010) and Hijmans (2012), we paid attention to the quality of the occurrence data and collected this vegetation data in May, June, and July 2012 by carefully revisiting the study area. The presence–absence of *M. latifolium* was recorded in five 1 m × 1 m subplots, one in the center and four at the corners of each 20 m × 20 m quadrat. It was considered present in a sample plot even if it was only detected in one of the five subsample plots. All trees with a diameter at breast height (dbh) larger than 7 cm were measured within each sample plot. At the same time, all shrubs were identified, each shrub species was counted, and the coverage percentage of each shrub species was recorded. We collected specimens of species which could not be identified in the field and identified them later in the Forest Faculty of Istanbul University Herbarium (ISTO) using the Flora of Turkey (Davis, 1965–1985; Davis, Mill, & Tan, 1988; Güner, Özhatay, Ekim, & Başer, 2000), and these specimens were deposited in the ISTO.

### 2.3 Environmental data

We selected environmental predictor variables used in previous SDM studies (Beaumont, Hughes, & Poulsen, 2005; Elith et al., 2006; Lobo et al., 2010; Warren & Seifert, 2011) and added fine‐scale topographic variables and monthly climatic data. Monthly climatic variables and bioclimatic variables were obtained from WorldClim database (http://www.worldclim.org). These data are a set of global climate layers with a spatial resolution of approximately 1 km^2^ (Hijmans, Cameron, Parra, & Albert, 2005).

In addition to climate data, this study used fine‐scale topographic variables obtained from terrain analysis that affect microclimate and other ecological processes. A total of 60 topographic variables such as slope, aspect, and curvature were derived from the ASTER DEM with a 30‐m resolution using the SAGA GIS terrain analysis functions (Conrad et al., 2015).

Solar radiation affects vegetation pattern, plant distribution, and growth by influencing near‐surface air temperature, soil temperature, and soil moisture within a region (Bennie, Huntleya, Wiltshirea, Hill, & Baxtera, 2008; Coblentz & Riitters, 2004). Continuous surface solar radiation data could be obtained from interpolation of weather station data, meteorological satellite data, and modeling solar radiation with GIS, and we preferred to use the latter method to calculate spatial solar radiation considering practical and widespread usage in natural studies. The “potential incoming solar radiation” module of SAGA GIS can be computed solar radiation for an instant time or a given day/week/month/year. Monthly solar radiation (direct solar radiation, diffuse solar radiation, total solar radiation, direct‐to‐diffuse solar radiation ratio, and the duration of solar radiation) was calculated taking the terrain shade effect into account using SAGA GIS (Conrad et al., 2015) under clear‐sky conditions.

There is a strong connection between bedrock composition and vegetation (Hahm, Riebe, Lukens, & Araki, 2014). A bedrock map was obtained from a 1/25.000 scale geological map prepared by the General Directorate of Mineral Research and Exploration (MTA). Bedrock type is the only categorical variable that was used in the study.

According to the literature (Davis & Stuart, 1984) and our observations in the field, *M. latifolium* requires specific habitat conditions and plant associations to survive and maintain its population. Therefore, some properties of trees and the shrub layer were used to determine the habitat of the plant and to estimate the potential distribution of the plant. The number of tree species per diameter class (8‐ 10.9, 11–19.9, 20–35.9, 36–51.9, 52–79,9 larger than 80 cm) of the 24 tree species existing in the sampling plots was calculated by the cumulative number of trees using the R package “vegclust” (De Cáceres, Font, & Olivia, 2010). The abundance‐cover value, richness, Shannon, Simpson, inverse Simpson, evenness, j evenness, and Berger indices of 73 species in shrub layer were also used.

To handle the effect of humans and livestock, we used proximity to the nearest residential area and the population of the area. The Euclidian distances of sample plots to the nearest residential areas were calculated using the “r.grow.distance” function on GRASS GIS (GRASS Development Team, 2014), and a raster output map was obtained. This variable was taken as it is indicating the impact of indirect human and domestic livestock grazing.

**Figure 3:**
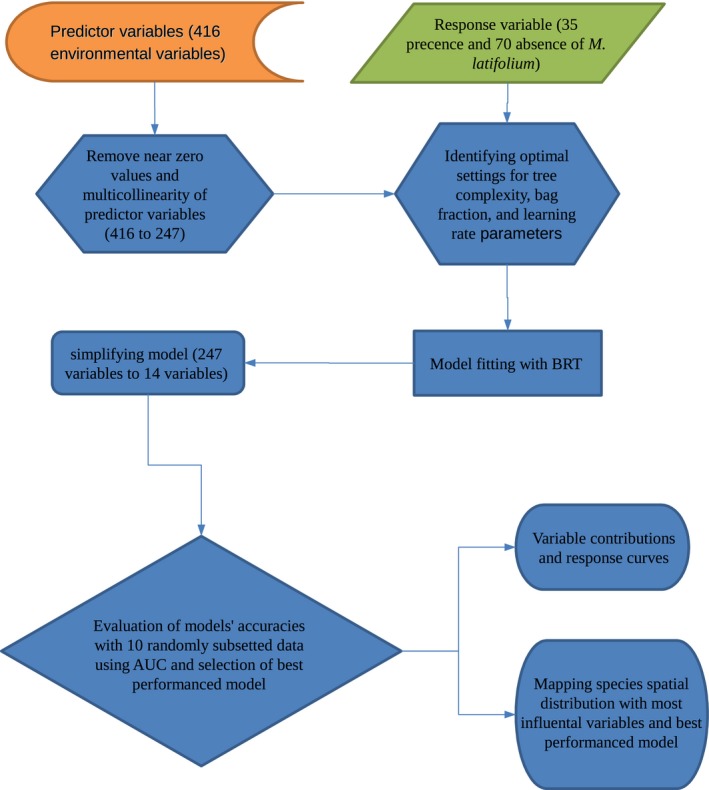
Schematic representation of the analysis steps used in the study

These direct, indirect, and resource variables obtained from GIS data layers used in the study were uploaded to the spatial point vector layer of sample plots using SAGA GIS software. Thus, a data matrix consisting of 416 aforementioned environmental variables (Table 1) and one response variable was prepared for further operations. Preprocessing was performed to achieve better model results before analyses were performed. First, zero‐variance predictors were removed for computing cost even though tree‐based models are impervious to this type of predictors (Kuhn & Johnson, 2013). Because we have more predictors than samples, we handled multicollinearity of DEM‐derived data by the simple five steps way suggested by Kuhn and Johnson (2013) instead of using a variance inflation factor. We did not do multicollinearity analysis for climatic variables because determining the true month of influential climatic variables and BRT is less sensitive than other methods for collinearity (Dormann et al., 2013). After preprocessing, 247 predictor variables remained for use in analysis. Figure 3 shows the study analyses process.

**Table 1:** Environmental variables used to model *Muscari latifolium* distribution in the study area (numbers of variable given in the parenthesis)

Variable (416)	Description	Source
Bioclim variables (19)	19 bioclimatic data calculated from temperature and precipitation	WorldClim database
Monthly climatic data (48)	Average monthly mean temperature, average monthly minimum temperature, average monthly maximum temperature, and average monthly precipitation	WorldClim database
Monthly solar radiation data (60)	Monthly total of diffuse, direct, and total solar radiation, and direct‐to‐diffuse ratio and duration of solar radiation (12*5 = 60)	Modeled from DEM with SAGA GIS
Topographic variables (60)	Topographic variables (such as slope, aspect, and curvatures)	Derived from DEM with SAGA GIS terrain analyses
Geology (1)	Bedrock type	MTA data
Biotic interaction variables (228)	CAPs of 24 tree species according to tree species at each diameter class of 6 (6*24 = 144)Cover values of 73 shrub species and 6 diversity indices (73 + 7 = 80)Distance to nearest residential area, man, woman, and total population of residential areas	Calculated from the study field dataCalculated from the study field dataCalculated with GRAS GIS and Turkish Statistical Institute data

### 2.4 Statistical methods

#### 2.4.1 BRTs

To specify the factors affecting the species’ distribution, we used BRTs (aka gradient boosting tree). BRT is a machine learning technique and has important advantages for tree‐based methods. Not only can it fit complex nonlinear relationships, but it can also handle interaction effects between predictors automatically (Elith et al., 2008). Detection of important relationships from large sets of predictor variables can be achieved (Barker, Cumming, & Darveau, 2014). Relatively poor predictive performance drawbacks of single tree models are tackled by BRT (Elith et al., 2008). Wisz et al. (2008) evaluated 12 algorithms for 46 species at three sample sizes (10, 30, and 100 records) and found that gbm was the best performing prediction algorithm at sample sizes 30 and 100.

#### 2.4.2 Model building

We used the dismo (Hijmans, Phillips, Leathwick, & Elith, 2015), gbm (Ridgeway, 2013), and raster (Hijmans & Etten, 2013) packages from the R statistical environment (R Development Core Team, 2014) for fit models, assessing relative contributions, making predictions, and mapping distribution. To prevent overfitting and determining user‐defined parameters used in BRTs, we evaluated tree complexity (1, 3, 5, 7), learning rate (0.1, 0.05, 0.01, 0.005, 0.001, 0.0005), and bag fraction (0.5, 0.75). Based on tenfold cross‐validation results, we selected 7 for tree complexity, 0.5 for bag fraction, and 0.005 for learning rate to achieve more than 1,000 trees suggested by Elith et al. (2008). Using these parameters, we built models with 105 *M. latifolium* incidences and a 247 environmental variable matrix. To reduce environmental noninformative variables, we simplified this model with the “gbm.simplfy” function (Elith & Leathwick, 2014) and removed 233 environmental variables. Simplification builds many models and drops unimportant variables using methods similar to backward selection in regression (Elith et al., 2008). Thus, 14 environmental variables (Table 2) remain to be used in the further steps.

**Table 2:** Most important variables selected according to final model performance

Variable	Description	Unit
Bio13	Precipitation of wettest month	Mm
Bio4	Temperature seasonality (standard deviation ×100)	°C × 100
Sunsetsep	Sunset of September	Time
Dir2difJul	Direct‐to‐diffuse insolation ratio in July	
Dir2difnov	Direct‐to‐diffuse insolation ratio in November	
Durinsnov	Duration of insolation in November	Hour
Dir2difMar	Direct‐to‐diffuse insolation ratio in March	
Mincur	Minimum curvature	
Northness	The degree to which a slope was northerly	
Bedrock	Bedrock type	
Qc1	Total number of *Quercus cerris*	Number
Pn4	Total number of *Pinus nigra* at diameter >36 cm	Number
Sortorm	*Sorbus torminalis* cover value (according to Van der Maarel 1979)	Percent
Growdist	Proximity to residential areas	Meter

#### 2.4.3 Model evaluation

We assessed the predictive performance of models using repeated subsampling processes. Ten random subdata sets were created from the entire data set. Each partition was created randomly selecting 70% (*n* = 74) presence/absence localities as training data, and the other 30% (*n* = 31) were selected as testing data. We used the area under a receiver operating characteristic curve (AUC) to evaluate the performance of each model. This metric is calculated from the receiver operating characteristic (ROC) plot that gives the false‐positive error rate (1‐specificity) on the x axis and the true positive rate (sensitivity) on the y axis (Franklin, 2009). The AUC is determined through summing the area under the ROC curve and taking the value between 0.5 and 1.0. Although Harrell (2001) states a threshold of 0.8 AUC value for models is necessary, Franklin (2009) states that a threshold of 0.5–0.7 AUC is considered poor, 0.7–0.9 AUC is considered moderate, and >0.9 AUC is considered high model performance. We created 10 models with tenfold cross‐validated train data sets using 14 environmental and one response variables. Then, predictive performance of these models was calculated on 10 replicate test data sets.

#### 2.4.4 Variable contributions and response curves

While assessing predictive performance for environmental variables, contribution to the model was also calculated over the 10 BRT model replicates. The most influential variables according to the sum of the relative influences of environmental variables in all models were selected and evaluated to determine the ecological requirements of the species.

#### 2.4.5 Spatial prediction

A final spatial prediction map was created from 13 of the 14 most important variables except *Sorbus torminalis* (L.) Crantz cover value. Potential spatial distribution of the *M. latifolium* prediction map was produced using a raster layer of these most important variables, and 1 of 10 models has the best prediction power. This map was produced with only part of the study area because not all of the forest survey data were up to date. These field survey data were interpolated with the regularized spline with tension method (Mitasova et al., 1995) which gives good prediction results for forest tree size attributes (Destan, Yılmaz, & Şahin, 2013).

## 3 Results

### 3.1 Model performance

The relationship between *M. latifolium* distribution and environmental variables was analyzed using 10 repeated BRTs models. These models’ accuracy was determined compared to test data sets. The overall average accuracy AUC value is 0.8. In total, 2 of the 10 models (m1, m2) were the most successful with an AUC value 0.93 (Table 3). Three models (Model 3, 4, and 9) gave AUC values that can be considered successful in the 0.80–0.9 range. While four models (m5, m6, m8, and m10) had AUC values between 0.70 and 0.8, only one model (m7) had an AUC value lower than 0.70 (0.68).

**Table 3:** Performance of 10 repeated boosted regression tree models

Model Number	ntree	calc.deviance	P	A	AUC	cor	max TPR + TNR at
1	1,550	0.65	13	18	0.93	0.78	0.60
2	1,050	0.75	11	20	0.93	0.73	0.21
3	2,000	0.92	12	19	0.87	0.63	0.44
4	5,700	0.97	11	20	0.85	0.55	0.52
5	1,150	0.71	10	21	0.76	0.44	0.41
6	1,350	0.75	11	20	0.71	0.42	0.51
7	1,100	0.83	12	19	0.68	0.29	0.40
8	1,200	0.72	8	23	0.74	0.39	0.63
9	1,550	0.46	8	23	0.80	0.51	0.48
10	1,600	0.56	11	20	0.72	0.38	0.42

### 3.2 Variable contributions and response curves

According to their relative contributions from 10 repeated BRT models, the seven most influential variables (the relative contribution average is greater than five) account for about the 70% of relative importance. Fourteen variables included in the final model in decreasing order of relative importance are ranked as follows: bedrock type (Bedrock), number of *Quercus cerris* L. (Qc1), precipitation of wettest month (Bio13), number of *P. nigra* (diameter >36 cm—Pn4), Northness, sunset September (Sunsetsep), *S. torminalis* cover value in shrub layer (Sortorm), proximity to residential areas (Growdist), temperature seasonality (Bio4), minimum curvature (Mincur), direct‐to‐diffuse insolation ratio in July (Dir2difJul), duration of insolation in November (Durinsnov), direct‐to‐diffuse insolation ratio in March (Dir2difMar), and direct‐to‐diffuse insolation ratio in November (Dir2difnov) (Table 4).

**Table 4:** Minimum, maximum, and average relative contributions (%) of the most influential environmental predictors calculated using tenfold cross‐validated BRT models of 10 random subsampled train data sets

Variable	Min	Max	Average
Bedrock	21.45	33.16	27.24
Qc1	8.61	16.99	12.58
Bio13	5.29	11.14	8.12
Pn4	3.90	10.09	7.15
Northness	2.78	11.97	6.17
Sunsetsep	2.17	8.01	5.37
Sortorm	2.75	8.23	4.99
Growdist	2.97	7.00	4.71
Bio4	2.12	9.16	4.58
Mincur	1.77	11.13	4.45
Dir2difjul	2.44	6.90	4.24
Durinsnov	1.26	10.39	4.10
Dir2difmar	0.75	6.64	3.40
Dir2difnov	1.41	4.79	2.89

Among those fourteen variables, bedrock type was the most influential variable on the distribution of *M. latifolium*. Six bedrock types are contained in 89 of 105 sample plots (85%) (Table 5). The numbers of sample plots where the species was absent on granodiorite, sandstone, Miocene‐aged andesitic tuff, Oligocene‐aged andesitic tuff, schist, and gneiss–mica‐schist bedrock types were 12, 12, 13, 9, 6, and 5, respectively, while the numbers of sample plots in which the species existed were 3, 2, 1, 8, 5, and 9, respectively (Table 5). *Muscari latifolium* was present more often than it was absent in plots containing only the gneiss–mica‐schist bedrock type (five absent, nine present).

**Table 5:** Presence/absence of *Muscari latifolium* on the six main bedrock types

Bedrock type	Absence	Presence
Granodiorite	12	3
Sandstone	12	2
Miocene‐aged andesitic tuff	13	1
Oligocene‐aged andesitic tuff	9	8
Schist	6	5
Gneiss–mica‐schist	5	9

Occurrences were closely associated with overstory trees. Qc1 was the second most important variable, and Pn4 was the fourth most important variable. The presence of the species in the field is closely associated with Qc1 and Pn4. Qc1 has a negative effect if the number of trees is less than five, and Pn4 also has a negative effect if the number of trees at this diameter class is less than three. We investigated these associations from the data set and found that according to the data set, *P. nigra* did not occur in six of 35 sample plots where *M. latifolium* was present while *Q. cerris* was not detected in 13 of 35 sample plots. Additionally, *Q. cerris* did not occur in two of six sample plots where *M. latifolium* was present, but *P. nigra* was absent. *P. nigra* did not occur in 23 of the 70 sample plots where *M. latifolium* was absent, and *Q. cerris* also did not occur in 45 of these plots. *Quercus cerris* did not occur in 14 of 23 sample plots in which both *M. latifolium* and *P. nigra* were absent.

**Figure 4:**
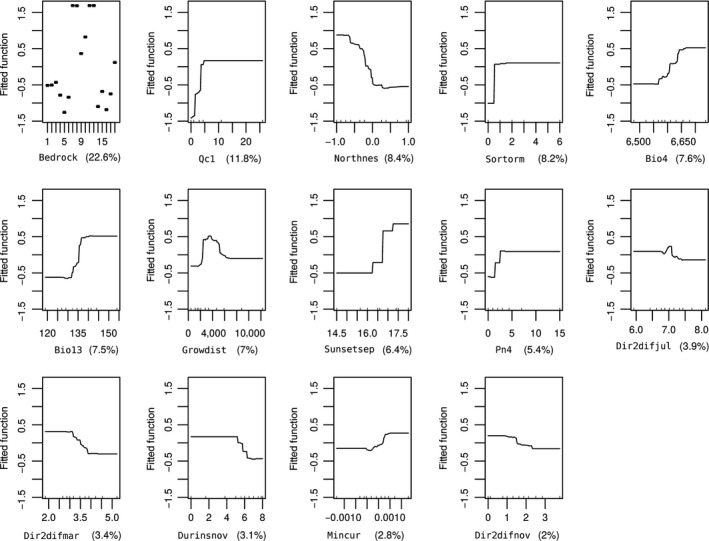
Partial dependence plots for the 14 most influential variables

The third most important variable was Bio13 (December is the wettest month in the study area). A minimum of 135 mm precipitation in December precipitation is associated for *M. latifolium* (Figure 4). The responses of *M. latifolium* to northness indicate that the species mostly occurs in the northwest and northeast. The Sortorm cover value is more than 1 in shrub layer which is positively associated with distribution of *M. latifolium*. *Muscari latifolium* is also positively affected when the distance to residential areas is between 2,000 and 6,000 m and temperature seasonality (standard deviation *100) (bio4) is >66°C. Mincur is another influential variable that has a positive effect when curvature increases. The occurrence of *M. latifolium* was also associated with the solar radiation variables. The distribution of *M. latifolium* is negatively affected when the average monthly duration of insolation in November exceeds 5 hr, the direct‐to‐diffuse insolation ratio of July is >7, the direct‐to‐diffuse insolation ratio of November is >1.5, and the direct‐to‐diffuse insolation ratio of March is >2.5, but influenced positively if the sunset of September is later than 17:00 local time.

### 3.3 Spatial prediction map

**Figure 5:**
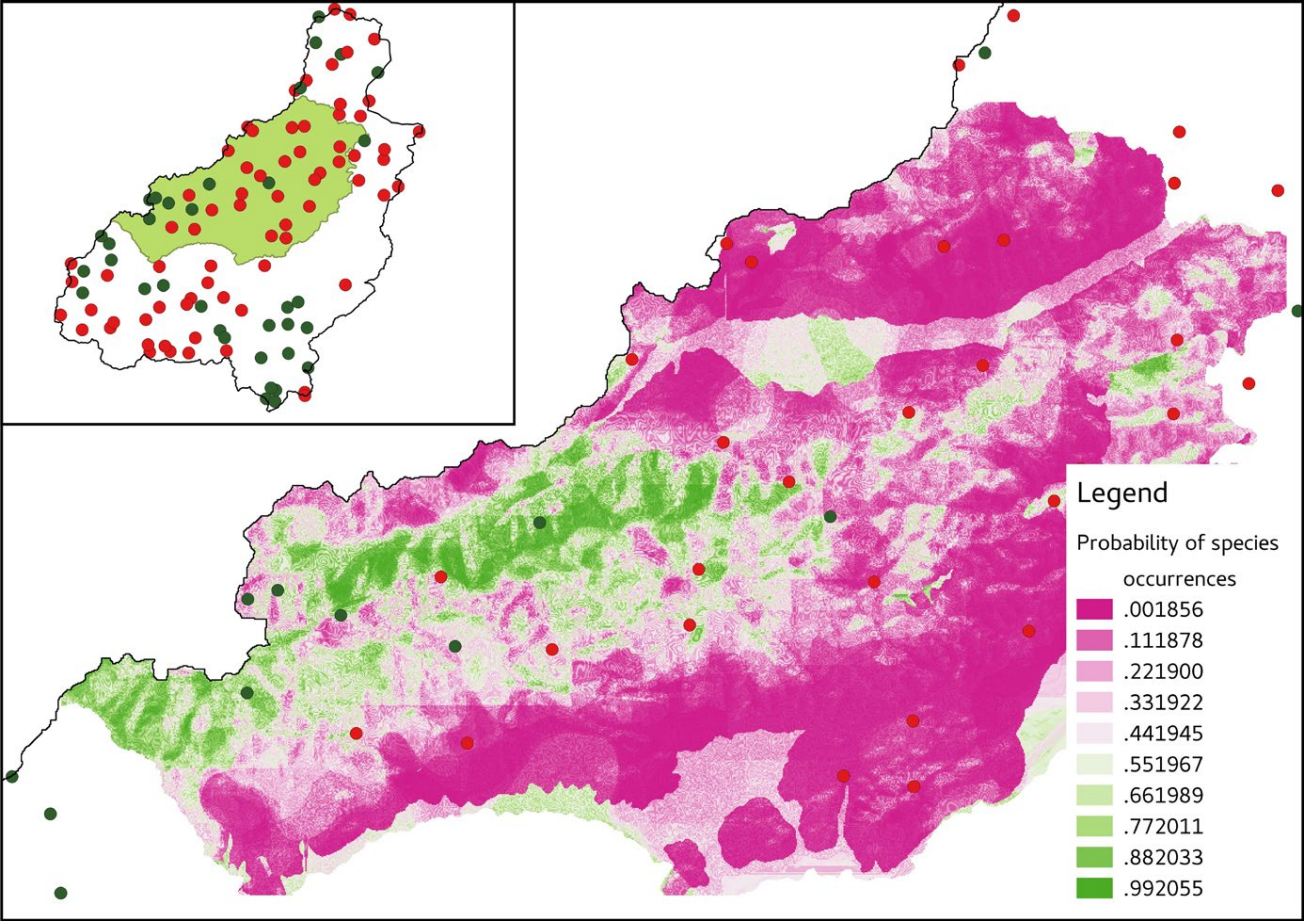
Potential spatial distribution map of *Muscari latifolium* obtained using the most influential variables upper left: green area shows the spatially predicted area within the whole study area

We also assessed the probability of presence/absence points of *M. latifolium* from a spatial prediction map (Figure 5). The spatial prediction map covered part of study area containing seven presence and 25 absence records of *M. latifolium*. The average and maximum probability value of presence was 0.64 and 0.97, respectively, and absence was 0.18 and 0.51, respectively.

## 4 Discussion

Plant distributions are limited not only when one environmental factor is less than the minimum required but also more than the maximum tolerance for a particular species (Billings, 1952). In this study, we used SDM to improve our understanding of the relationship between *M. latifolium* distribution and environmental factors. Species distribution modeling provides us valuable information that is useful in management and effective conservation strategies, particularly for rare and endemic plant species. Additionally, assessing the potential impact of climate change on species distribution (Thuiller, Brotons, Araújo, & Lavorel, 2004) that can be projected by SDM allows the development of strategies for sustainable management. The BRTs modeling approach applied here gives a realistic picture of a potential distribution of *M. latifolium* in the Gönen Dam watershed that can be used for these aims.

Our results showed that the fine‐scale distribution of *M. latifolium* is controlled mainly by geological, climatic, topographic, solar radiation, and biotic variables at the study area. Analysis performed on these biotic and abiotic variables showed that there were 14 factors that mostly influenced the species’ distribution (Table 4). These variables create the most favorable growth environment for this species.

Bedrock type is proved to be the most influential variable on the distribution of *M. latifolium*. This is because bedrock is the main factor affecting soil properties such as climate, relief, altitude, and living organisms (Beieler, 1975; Hartmann & Moosdorf, 2012). Moreover, bedrock has an important role explaining differences in vegetation (Hahm et al., 2014). This is mainly related to the fact that soil is developed from different bedrocks in different textures, which may affect the species’ distribution. Sandy soils, where *M. latifolium* is present, were formed mostly from granodiorite and sandstone. On the other hand, clay soils, where *M. latifolium* is absent, were derived from schist and mica schist.

Several climatic variables are also proved important for the distribution of *M. latifolium*. The increase in temperature seasonality had a positive effect on the habitat suitability of *M. latifolium,* while the species is unable to tolerate lower temperature seasonality. This is likely related to seasonal thermoperiodicity which is the most important factor controlling growth, development, and flowering in geophytes most of which need warm–cold–warm period to their annual life cycle (Khodorova & Boitel‐Conti, 2013). Tolerances of individual species for extreme seasonality are generally conserved across phylogeny. Therefore, temperature seasonality can be used to accurately predict the range limits of species in SDMs (Wiens, Graham, Moen, Smith, & Reeder, 2006). Precipitation during the wettest month (December in the study area) is thought to be a limiting factor of *M. latifolium* to survive and maintain its population when it is <135 mm. According to Doussi and Thanos (2002), *Muscari* seeds need a rainy season in early winter to germinate in the Mediterranean climate. December precipitation may affect the distribution of *M. latifolium* by influencing its germination.

Solar radiation appears to be an important factor on *M. latifolium* distribution particularly in March, July, September, and November. The sunset in September later than 17:00 has a positive effect on the distribution of *M. latifolium*. Although bulbous plants seem to be dormant in autumn and winter, active developmental processes continue in this period using reserves which are in the underground organ and are also affected by temperature conditions (Khodorova & Boitel‐Conti, 2013). Therefore, *M. latifolium* might require more exposure to sunlight in September, whereas it exists only as bulb and seed below soil in this period. On the other hand, the increment of duration of insolation in November and the increment of direct‐to‐diffuse insolation ratio in March and November have a negative effect on the distribution of the species. Doussi and Thanos (2002) indicated that exposure to daylight caused a decrease in germination rate in some *Muscari* species even if it led to the emergence of secondary seed dormancy. November solar radiation, March solar radiation, and the wettest month (December) precipitation variables directly affect plant germination; therefore, they are noteworthy factors affecting the distribution of the plant in the area. The direct‐to‐diffuse insolation ratio in July might also be associated with maturation process and spreading of seeds.

Northness is also an influential topographic variable on the distribution of *M. latifolium*; it mostly occurs in the northwest and northeast aspects in the study area. Northness is an important explanatory variable on a fine‐scale because it refers to the solar radiation contrast between north and south faces and it is a limiting factor on the growth period along north faces related to snow cover duration (Lasseur, Joost, & Randin, 2006). Minimum curvature, another topographic influential variable, has a positive effect when curvature increases. To investigate this relationship, we visually interpreted the *M. latifolium* occurrence map draped over the minimum curvature map and most of the presence was detected at the steep slope convergence areas, mainly on spurs and ridges that have relatively higher minimum curvature values. The minimum curvature is likely to affect soil properties in such a way that it is favorable for the establishment of *M. latifolium* (Shary, Larisa, Sharaya, & Mitusov, 2002).

The occurrence of the species in the study area was closely associated with biotic variables characterized by overstory tree species, particularly *P. nigra* and *Q. cerris*, the coverage value of *S. torminalis* in the shrub layer, and proximity to residential areas. *Muscari latifolium* occurs in, pure *P. nigra* forests, mixed *P. nigra* and *Quercus* sp. forest and oak‐dominant mixed deciduous forest. This might be explained by the influence of forest overstory on the herb layer through modifications of resource availability (light, water, and soil nutrients) (Barbier, Gosselin, & Balandier, 2008). Additionally, López, Larrea‐Alcázar, and Ortuño (2009) found that several herbaceous species are associated exclusively with the shrub undercanopy and he suspected that this is caused by facilitation. The proximity of the sampling plots to settlement areas has positive effects only when the plots are between 2,000 and 6,000 m away. The relationship between the distribution of *M. latifolium* and settlement areas is very complex and hard to explain. The negative effect observed when the sampling plots are closer than 2,000 m to a settlement may be explained as a result of grazing. Ruminants grazing was observed in some areas during the field surveys. In the same way, Louhaichi, Salkini, and Petersen (2009) determined that the number of geophyte species and the percentage of geophytes in a grazed area were dramatically lower than in ungrazed areas in semiarid Mediterranean Ecosystems. Also Chaideftou, Thanos, Bergmeier, Kallimanis, and Dimopoulos (2009) stated that the seeds of many species (such as *Muscari neglectum*) that exist in the vegetation of grazed areas could not be found in the seed soil bank of the Mediterranean oak forest. Grazing affects species distribution and composition adversely, and more pressure might contribute decline of the species. Although “r.growdist” function of GRASS GIS software (GRASS Development Team, 2014) gives the proximity to the settlement area, calculating it using a function that takes the terrain into account may give better results. Biotic variables are important to understand the fine‐scale distribution and abundance of species (Meineri, Skarpaas, & Vandvik, 2012) and improve both the fit and the predictive power of distribution models (Pellissier et al., 2010).

Our model establishes the importance of geologic, climatic, topographic, solar radiation, and biotic variables to the occurrence of *M. latifolium*. Due to a lack of regional information on *S. torminalis* cover and the lack of a raster map, this variable was removed from the spatial prediction map model of *M. latifolium*. Biotic variables’ data related to vegetation can be obtained from remote sensing images and can increase the accuracy of models (Swatantran et al., 2012; Wilson, Sexton, Jobe, & Haddad, 2013). However, it is not easy to obtain the cover value of *S. torminalis* with high accuracy from remote sensing images. Nevertheless, this variable provides an important contribution to the knowledge of the habitat requirements and ecological characteristics of the species. Moreover, the potential distribution map of *M. latifolium* obtained in this study provides a good basis for the management, conservation, and climate change strategies of this species in the study area, although it did not include *S. torminalis* cover values.

Ideally, ecologically most relevant variables for a species should be used within SDMs. However, when studied species is endemic and priori information is unavailable, the number of variables that could potentially be used to predict species distribution is almost infinite and has collinearity. Hence, variable selection becomes an important issue that BRTs can bring a solution to. Other important issue that we paid attention in the current study is the true absences that provide potentially relevant information on species ecology (Thuiller et al., 2004).

In conclusion, this study provides significant contribution to the knowledge of the habitat requirements and ecological characteristics of *M. latifolium*. The distribution of this species is explained by a combination of biotic and abiotic factors. The information obtained in this study can be used to support management, conservation, and, if needed, restoration programs for this species. *M. latifolium* was listed in the low critical (LC) category by Ekim et al. (2000) and endangered (EN) category by Özhatay and Özhatay (2005). The red list category of this fragmented and limited distributed species needs to be revised, and this potential distribution map may help in this effort. The absence of some biotic factors, such as dispersal limitations, and overstory trees, prevent the model from being more robust. However, bioclimatic variables and solar radiation variables were detected as influential factors that affect the distribution of *M. latifolium* and can provide valuable information about ecological characteristics of *M. latifolium*. The BRT model used in the study has reasonable model performance and simplifying mechanism that reduce uninformative variables easily.

## Conflict of Interest

None declared.
